# Efficacy of moxibustion in diarrhea-predominant irritable bowel syndrome model rats: a systematic review and meta-analysis

**DOI:** 10.3389/fbioe.2023.1309661

**Published:** 2023-12-15

**Authors:** Xiang Rao, Yu Xing, Changchun Ji, Jixing Guo, Hong-Bao Li, Chaoju Xie, Juan Bai, Xuejiao Wang, Ying Li, Jingyu Zhao

**Affiliations:** ^1^ College of Acupuncture and Moxibustion, Shaanxi University of Traditional Chinese Medicine, Xianyang, China; ^2^ Department of Acupuncture and Moxibustion, Shaanxi Hospital of Traditional Chinese Medicine, Xi’an, China; ^3^ Department of Physiology and Pathophysiology, Xi’an Jiaotong University School of Basic Medical Sciences, Xi’an, China; ^4^ Department of Anesthesiology, Center for Brian Science, the First Affiliated Hospital of Xi’an Jiaotong University, Xi’an, China; ^5^ Department of Acupuncture and Moxibustion, Xi’an Hospital of Traditional Chinese Medicine, Xi’an, China

**Keywords:** moxibustion, irritable bowel syndrome, systematic review, meta-analysis, animal model, rats

## Abstract

**Objective:** To systematically evaluate the efficacy of moxibustion in diarrhea-predominant irritable bowel syndrome (IBS-D) model rats.

**Methods:** A comprehensive search was conducted in the China National Knowledge Infrastructure, WanFang Data, VIP, PubMed, Embase, and Web of Science databases from their inception to June 30, 2023. Relevant animal experiments investigating moxibustion for treating IBS-D in model rats were included. Two independent researchers screened the literature, extracted data, and evaluated the risk of bias in the selected studies. The meta-analysis was performed using RevMan 5.3 software.

**Results:** In total, 21 animal studies comprising 680 model rats were included. The meta-analysis results demonstrated that moxibustion enhanced the threshold capacity of the abdominal withdrawal reflex (AWR) [standardized mean difference (SMD) = 1.84; 95% confidence interval (CI): 1.09, 2.60; *p* < 0.00001], ameliorated the rate of loose stool (SMD = −4.03; 95% CI: –5.76, −2.30; *p* < 0.00001), and decreased the colon 5-hydroxytryptamine (SMD = −3.67; 95% CI: –5.33, −2.01; *p* < 0.00001), serum interleukin-1β (SMD = −3.24, 95% CI: –4.06, −2.41; *p* < 0.00001), serum tumor necrosis factor-α (SMD = −2.35, 95% CI: –4.12, −0.58; *p* < 0.00001), and serum substance P (SMD = −5.14, 95% CI: –8.45, −1.83; *p* = 0.002) concentrations. Moxibustion did not affect the blood calcitonin gene-related peptide level compared to the blank model group (*p* = 0.15).

**Conclusion:** Moxibustion modulated the brain-gut interaction, reduced visceral hypersensitivity, inhibited intestinal inflammation, and regulated the immune balance, improving the rate of loose stool and increasing the AWR threshold capacity in IBS-D model rats, achieving good analgesic and antidiarrheal effects. However, these conclusions require further validation due to limitations in the quantity and quality of the included studies.

## 1 Introduction

Irritable bowel syndrome (IBS) is a prevalent functional gastrointestinal disorder characterized by primary symptoms of abdominal pain, bloating, or abdominal discomfort, often associated with alterations in bowel habits, such as changes in bowel frequency and stool consistency (Liu and Liu, 2021). Epidemiological investigations indicate a global IBS prevalence of approximately 11.2% (Enck et al., 2016), which has escalated over time. Among its subtypes, diarrhea-predominant irritable bowel syndrome (IBS-D) is the most prevalent, accounting for approximately 40.83% of all IBS cases (Sun H. et al., 2023). IBS-D significantly affects the physical and mental wellbeing of the affected individuals, diminishing their quality of life and work capacity and imposing substantial healthcare economic burdens on society.

The pathophysiological mechanisms underlying IBS-D remain unclear but have been closely associated with many factors, including the brain-gut axis, visceral hypersensitivity, intestinal inflammation, and immune responses. Contemporary medicine often relies on symptomatic supportive therapies, such as antidiarrheal agents, antispasmodics, and antibiotics (Lembo et al., 2022). However, their efficacy for IBS-D is inconclusive, and prolonged use may lead to adverse effects, including constipation, dizziness, and nausea (Vasant et al., 2021). Moxibustion therapy, guided by the principles of traditional Chinese medicine’s meridian and acupoint theory, offers a non-pharmacological intervention devoid of drug-related adverse effects. Stimulating specific acupoints on the body’s surface harnesses the body’s endogenous healing capabilities to prevent and treat diseases. Clinical research has indicated that moxibustion ameliorates IBS-D symptoms, including diarrhea and abdominal pain (Chen G. G. et al., 2021; Wang et al., 2021; Zhao et al., 2023), and several systematic reviews have assessed the clinical effectiveness and safety of moxibustion therapy for IBS-D, supporting its beneficial effects (Huang et al., 2018; He et al., 2019; Ye et al., 2019).

Evidence-based studies in animal models play a critical role in establishing the safety and effectiveness of intervention strategies, especially in elucidating the mechanisms and key pathways involved in their therapeutic actions. Several animal experiments have suggested that moxibustion modulates levels of various markers in IBS-D rats, including 5-hydroxytryptamine (5-HT), tumor necrosis factor-α (TNF-α), calcitonin gene-related peptide (CGRP), and interleukin-1β (IL-1β). However, the outcome measures employed in these studies vary significantly, and some results exhibit notable discrepancies, indicating a lack of systematic investigation. A systematic review of the mechanisms underlying moxibustion’s therapeutic effects in IBS-D can provide insight into its potential treatment mechanisms and clinical efficacy, offering a foundational basis and guidance for further mechanistic research and preventing the wastage of experimental resources. Simultaneously, a systematic review of the quality of past research designs in moxibustion therapy for IBS-D can encourage future studies to adhere to quality assessment standards when designing and executing experiments, enhancing the quality of subsequent animal experimental studies (Liu et al., 2020). Therefore, this study systematically evaluated the efficacy of moxibustion therapy in treating IBS-D in model rats, further exploring the biological mechanisms underlying the effectiveness of moxibustion. Ultimately, we aimed to elucidate moxibustion’s role in IBS-D.

## 2 Methods

### 2.1 Inclusion criteria

The inclusion criteria were: (a) Research type: Animal experiments designed with randomized controlled trials (RCT) published in Chinese or English; (b) Study object: Experimental IBS-D model rats, regardless of modeling methods, age, weight, or gender. (c) Interventions: Experimental group: Involved any form of moxibustion. There were no restrictions on the selection of points, intervention duration, and treatment course. Control group: Blank control group–IBS-D rats; (d) Outcomes indicators: The rate of loose stool, the abdominal withdrawal reflex (AWR) threshold capacity, and the 5-HT, IL-1β, TNF-α, substance P (SP), and CGRP concentrations.

### 2.2 Exclusion criteria

The exclusion criteria were: (a) non-RCT designs; (b) rats with other coexisting diseases; (c) duplicate publications; (d) inaccessible full text; (e) conference papers, reviews, and studies with unavailable data.

### 2.3 Search strategy

Computer searches were conducted in the China National Knowledge Infrastructure (CNKI), Wanfang, VIP, PubMed, Embase, and Web of Science databases from their inception to June 30, 2023, to identify animal experiments involving moxibustion treatment of IBS-D model rats. Distinct search strategies were employed for the various databases, combining subject and free-text terms. The following search terms and their synonyms were used in the literature search: irritable bowel syndrome, IBS, irritable colon, spastic colon, irritable bowel, functional bowel, colonic disease, gastrointestinal syndrome, moxibustion, separated moxibustion, heat sensitive point moxibustion, rat, and rats.

### 2.4 Literature screening and data extraction

Two independent researchers screened the literature and extracted the data (with cross-validation) based on the inclusion and exclusion criteria, outcome measures, and research objectives. Disagreements were resolved through discussion, and if necessary, a third party was consulted for decision-making assistance. NoteExpress V3.6.0 (Aegean Software Corp., Beijing, China) was used for literature management. For duplicate publications, studies with complete data were retained. During the literature screening process, titles were initially reviewed to exclude obviously unrelated articles. Subsequently, the abstracts and full texts were examined to determine the study’s eligibility. When needed, the first or corresponding authors were contacted via email to obtain essential information that was unclear but crucial for this study. The data extraction process included the following information: basic details of included studies (first author, publication year, and journal), baseline characteristics (sample sizes, age, sex, quality, and modeling methods for each group), intervention details (acupuncture point selection and intervention duration), outcome measures, and key elements for assessing bias risk.

### 2.5 Quality assessment and risk of bias

The SYRCLE animal experiment bias tool was used to assess the risk of bias (Hooijmans et al., 2014; Tao et al., 2019), which comprises ten entries related to six types of bias: (1) selection bias (sequence generation, baseline characteristics, allocation concealment); (2) performance bias (random housing and blinding of animal placement); (3) detection bias (random outcome assessment and blinding); (4) attrition bias (incomplete outcome data); (5) reporting bias (selective outcome reporting); and (6) other sources of bias. Scores of “yes,” “no,” and “unclear” represent low, high, and uncertain bias risks, respectively.

### 2.6 Data analysis

The included studies were statistically analyzed using RevMan 5.3 software (Cochrane, London, UK). The measures were analyzed using the mean differences or standardized mean differences (SMDs) and 95% confidence intervals (CIs). The heterogeneity of the included studies was tested using *I* ^2^ values; a *p*-value of >0.10 and an *I* ^2^ value of <50% indicated acceptable heterogeneity and homogeneous study results. In this case, a fixed-effect model was used for the meta-analysis. If the *p*-value was ≤0.10 and the *I* ^2^ value was ≥50%, the study results were considered heterogeneous, and the sources were analyzed using sensitivity or subgroup analyses to identify the factors causing heterogeneity. If heterogeneity could not be eliminated, the random-effects model was used for the meta-analysis (Zhang, 2020). Publication bias was assessed using funnel plots, with asymmetric graphs indicating the presence of publication bias and *vice versa*.

## 3 Results

### 3.1 Literature screening

**FIGURE 1:**
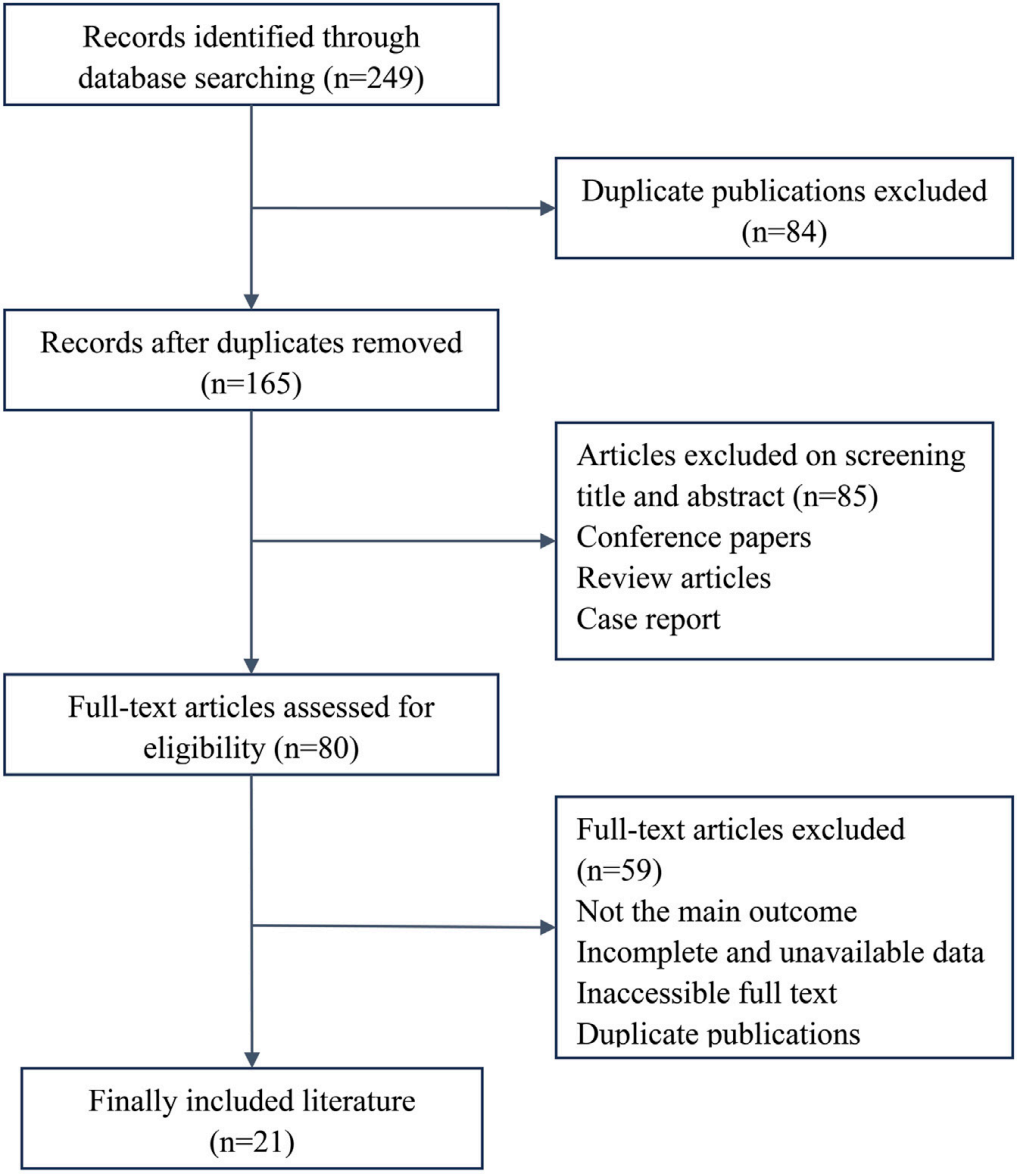
Flow diagram of the meta-analysis study selection process.

The preliminary database search yielded 249 papers (39 from CNKI, 96 from Wanfang, 26 from VIP, 26 from PubMed, 28 from Embase, and 34 from Web of Science), including 161 Chinese-language and 88 English-language papers. In total, 84 duplicates were excluded, resulting in 165 articles from the primary screening. The titles and abstracts were screened, excluding the studies with inconsistent study subjects and interventions, resulting in 80 articles. Finally, the full text was read to exclude non-RCTs, duplicate publications, and studies with unavailable data, resulting in 21 RCTs in the final analysis (Figure 1) (Zhou, 2008; Zhou et al., 2009; Du, 2011; Zhang, 2012; Zhao, 2013; Zhang et al., 2017a; Zhang et al., 2017b; Wu, 2018; Fu et al., 2019; Jianan, 2019; Zheng et al., 2019; Tong, 2020; Wang et al., 2020; Ch et al., 2021; Shi et al., 2021; Wan, 2021; Wang, 2021; Wu et al., 2021; Li, 2022; Liao et al., 2022; Zhang et al., 2022).

### 3.2 Basic characteristics and risk of bias

Tables 1, 2 present the basic characteristics of the included studies and the risk of bias assessment results, respectively.

**TABLE 1:** Basic characteristics of the included studies.

Study	Animal characteristics	Animal models	Animal numbers T/C	Intervention	Acupoint	Duration (days)	Outcome measures
Zhang et al. (2022)	SD rats (male)	Senna leaf decoction gastric infusion + chronic unpredictable stress	6/6	Moxibustion	Mingmen (DU4)	14	6, 7
	190 ± 10 g						
Liao et al. (2022)	SD rats (any sex)	Maternal-infant separation + acetic acid stimulation + chronic restraint	12/12	Moxibustion	Tianshu (ST25), Shangjuxu (ST37)	7	3, 6, 7
Li (2022)	SD rats (male)	Senna leaf decoction gastric infusion + chronic restraint	6/6	Moxibustion	Mingmen (DU4)	14	1
	190 ± 10 g						
Wang (2021)	SD rats (male)	Ice acetic acid enema + chronic restraint	10/10	Moxibustion	Tianshu (ST25)	7	4
	180 ± 10 g						
Wan (2021)	SD rats (male)	Ice acetic acid enema + chronic restraint	10/10	Moxibustion	Tianshu (ST25)	7	2, 5
	120 ± 20 g						
Wu et al. (2021)	SD rats (male)	Senna leaf decoction gastric infusion + chronic restraint	10/10	Moxibustion	Tianshu (ST25), Shangjuxu (ST37)	7	1, 2
	220 ± 14 g						
Ch et al. (2021)	SD rats (male)	Chronic unpredictable stress	12/12	Moxibustion	Tianshu (ST25), Shangjuxu (ST37)	14	2
	200 ± 20 g						
Shi et al. (2021)	Wistar rats (male)	Chronic unpredictable stress	8/8	Moxibustion	Tianshu (ST25), Shangjuxu (ST37)	7	7
	150 ± 20 g						
Tong (2020)	SD rats (male)	Senna leaf decoction gastric infusion + chronic restraint	10/10	Moxibustion	Tianshu (ST25), Shangjuxu (ST37)	7	1, 2, 4, 5
	225 ± 25 g						
Wang et al. (2020)	SD rats (male)	Senna leaf decoction gastric infusion + chronic restraint	8/8	Moxibustion	Tianshu (ST25), Shangjuxu(ST37)	7	1, 2
	225 ± 25 g						
Zheng et al. (2019)	Wistar rats (male)	High lactose diet + mild stress + peripheral sensitization method	15/15	Heat-sensitive moxibustion	Zusanli (ST36)	14	1, 6
	280 ± 30 g				Shenque (RN8)		
Fu et al. (2019)	SD rats (male)	Chronic unpredictable stress	8/8	Moxibustion	Mingmen (DU4)	14	1
	230 ± 10 g						
Jianan (2019)	SD rats (male)	Acetic acid enema + restraint stress	12/12	Herb-partition moxibustion	Ganshu (BL18), Pishu (BL20), Zusanli (ST36), Zhangmen (LR13), Qimen (LR14)	14	1, 3
	180 ± 10 g						
Wu (2018)	SD rats (male)	Senna leaf decoction gastric infusion + chronic restraint	10/10	Moxibustion	Tianshu (ST25), Shangjuxu (ST37)	7	2, 4, 5
	195 ± 20 g						
Zhang et al. (2017a)	SD rats (male)	Chronic unpredictable stress	8/8	Moxibustion	Mingmen (DU4)	14	1
	230 ± 10 g						
Zhang et al. (2017b)	SD rats (male)	Chronic unpredictable stress	8/8	Moxibustion	Mingmen (DU4)	14	7
	230 ± 10 g						
Zhao (2013)	SD rats (male)	Maternal-infant separation + direct colon balloon dilation method	10/10	Moxibustion	Tianshu (ST25)	10	3
Zhang (2012)	SD rats (male)	Senna leaf decoction gastric infusion + cold water stress	10/10	Spreading moxibustion	Ganshu (BL18) to Dachangshu (BL25), Zhongwan (RN11) to Guanyuan (RN4)	14	1, 3
	200 ± 20 g						
Du (2011)	Wistar rats (female)	Intraluminal chemical stimulation	7/7	Moxibustion	Zusanli (ST36)	6	7
	200 ± 20 g						
Zhou et al. (2009)	SD rats (male)	Maternal-infant separation + direct colon balloon dilation method	9/9	Herb-partition moxibustion	Tianshu (ST25)	7	3
Zhou (2008)	SD rats (male)	Maternal-infant separation + direct colon balloon dilation method	6/6	Herb-partition moxibustion	Tianshu (ST25)	7	3

Notes: 1: Abdominal withdrawal reflex; 2: The rate of loose stool; 3: 5-hydroxytryptamine level in the colon; 4: Interleukin-1β level in the serum; 5: Tumor necrosis factor-α level in the serum; 6: Substance P level in the serum; 7: Calcitonin gene-related peptide level in the blood.

Abbreviations: SD, Sprague-Dawley.

**TABLE 2:** The risk of bias assessment.

Study	1	2	3	4	5	6	7	8	9	10
Zhang et al. (2022)	U	Y	U	Y	U	U	Y	Y	Y	Y
Liao et al. (2022)	U	Y	U	Y	U	U	Y	Y	Y	Y
Li (2022)	U	Y	U	Y	U	U	Y	Y	Y	Y
Wang (2021)	U	U	U	Y	U	U	Y	Y	Y	Y
Wan (2021)	Y	U	U	Y	U	U	Y	Y	Y	Y
Wu et al. (2021)	Y	U	U	Y	U	U	Y	Y	Y	Y
Ch et al. (2021)	Y	U	U	Y	U	U	Y	Y	Y	Y
Shi et al. (2021)	U	U	U	Y	U	U	Y	Y	Y	Y
Tong (2020)	Y	U	U	Y	U	U	Y	Y	Y	Y
Wang et al. (2020)	Y	U	U	Y	U	U	Y	Y	Y	Y
Zheng et al. (2019)	Y	U	U	Y	U	U	Y	Y	Y	Y
Fu et al. (2019)	Y	U	U	Y	U	U	Y	Y	Y	Y
Jianan (2019)	U	Y	U	Y	U	U	Y	Y	Y	Y
Wu (2018)	Y	U	U	Y	U	U	Y	Y	Y	N
Zhang et al. (2017a)	Y	U	U	Y	U	U	Y	Y	Y	Y
Zhang et al. (2017b)	Y	U	U	Y	U	U	Y	Y	Y	Y
Zhao (2013)	U	U	U	Y	U	U	Y	Y	Y	Y
Zhang (2012)	U	U	U	Y	U	U	Y	Y	Y	Y
Du (2011)	U	U	U	Y	U	U	Y	Y	Y	Y
Zhou et al. (2009)	U	Y	U	Y	U	U	Y	Y	Y	Y
Zhou (2008)	U	Y	U	Y	U	U	Y	Y	Y	Y

Note: 1. Sequence generation; 2. Baseline characteristics; 3. Allocation concealment; 4. Random housing; 5. Blinding of animal placement; 6. Random outcome assessment; 7. Blinding; 8. Incomplete outcome data; 9. Selective outcome reporting; 10. Other bias.

Abbreviations: Y, low risk of bias; N, high risk of bias; U, uncertain risk of bias.

### 3.3 Meta-analysis results

#### 3.3.1 AWR threshold capacity

**FIGURE 2:**
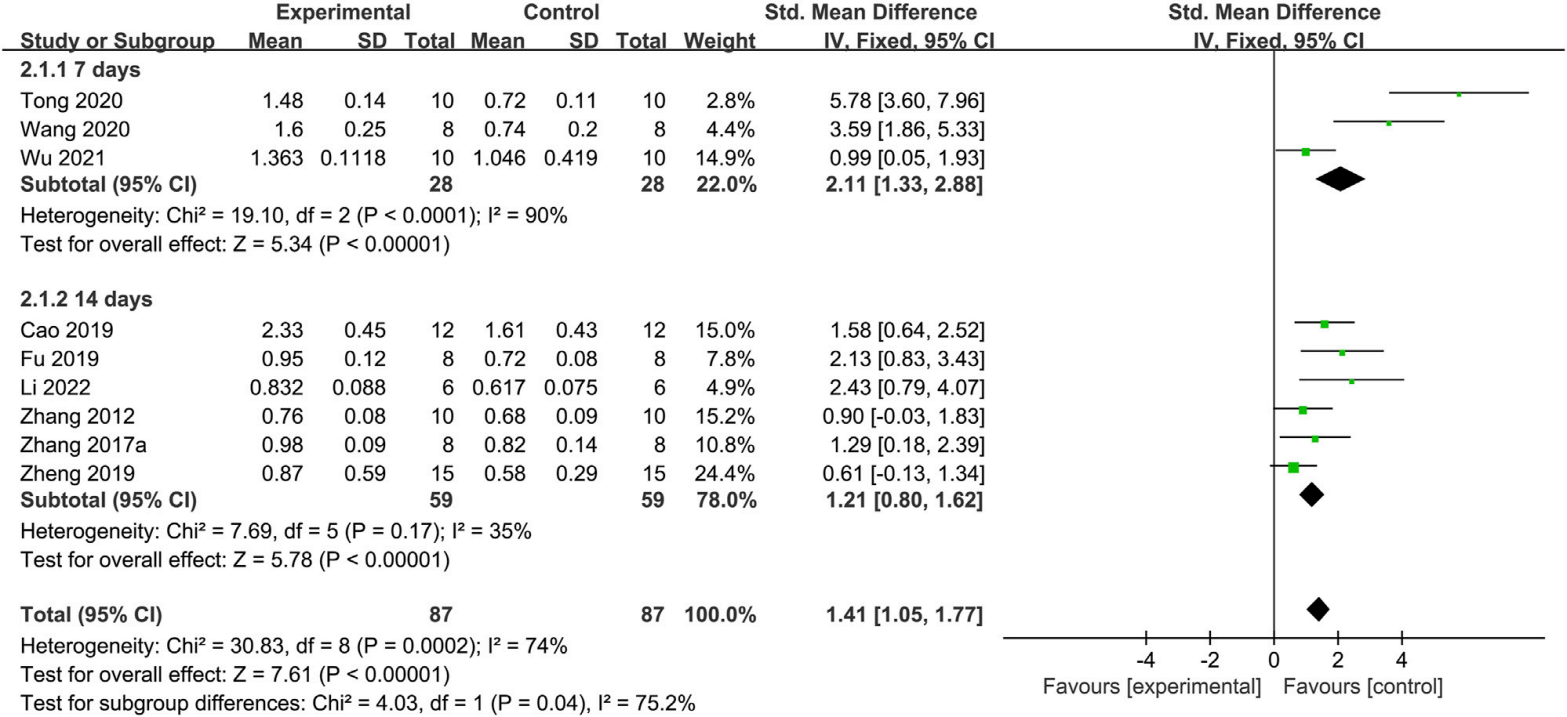
Forest plot analysis of the threshold capacity of abdominal withdrawal reflex. CI, Confidence interval; SD, Standard deviation.

Nine studies reported the AWR threshold capacity (Figure 2) (Zhang, 2012; Zhang et al., 2017a; Fu et al., 2019; Jianan, 2019; Zheng et al., 2019; Tong, 2020; Wang et al., 2020; Wu et al., 2021; Li, 2022). The meta-analysis results indicated significant heterogeneity (*I* ^*2*^ = 74%, *p* = 0.0002). Thus, a random-effects model was used to pool the effect sizes, demonstrating that moxibustion significantly increases the AWR threshold capacity (SMD = 1.84; 95% CI: 1.09, 2.60; *p* < 0.00001). A sensitivity analysis was performed, suggesting that treatment duration affected the heterogeneity. Thus, the studies were divided into two subgroups based on the treatment duration. Heterogeneity testing indicated that the 14-day treatment subgroup had low heterogeneity (*I* ^*2*^ = 35%) and could be analyzed using a fixed-effects model. In contrast, the 7-day treatment subgroup had high heterogeneity (*I* ^*2*^ = 90%), indicating that different moxibustion treatment durations affect the threshold capacity. These results support the conclusion that moxibustion increases the AWR threshold capacity in IBS-D model rats.

#### 3.3.2 The rate of loose stool

**FIGURE 3:**
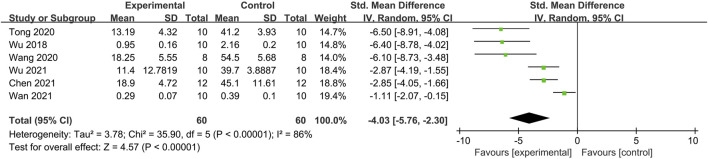
Forest plot analysis of the rate of loose stool. CI, Confidence interval; SD, Standard deviation.

Six studies reported the rate of loose stool (Figure 3) (Wu, 2018; Tong, 2020; Wang et al., 2020; Ch et al., 2021; Wan, 2021; Wu et al., 2021). The meta-analysis showed significant heterogeneity (*I* ^*2*^ = 86%, *p* < 0.00001). A random-effects model was used to pool the effect size, yielding an SMD of −4.03 (95% CI: –5.76, −2.30; *p* < 0.00001), indicating a significant difference. The sensitivity analysis did not identify the source of heterogeneity. These results suggest that moxibustion improves the rate of loose stool in the IBS-D rat model.

#### 3.3.3 5-HT

**FIGURE 4:**
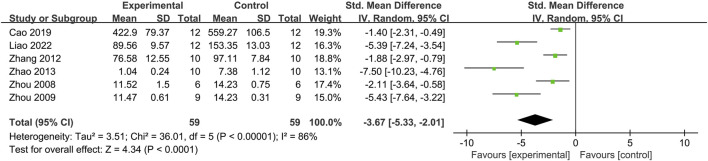
Forest plot analysis of the colon 5-hydroxytryptamine concentration. CI, Confidence interval; SD, Standard deviation.

Six studies investigated the 5-HT concentration (Figure 4) (Zhou, 2008; Zhou et al., 2009; Zhang, 2012; Zhao, 2013; Jianan, 2019; Liao et al., 2022). The meta-analysis revealed significant heterogeneity (*I* ^*2*^ = 81%, *p* < 0.0001). Therefore, a random-effects model was used to pool the effect size, resulting in an SMD of −3.67 (95% CI: –5.33, −2.01; *p* < 0.00001), indicating a significant difference. The sensitivity analysis did not identify the source of heterogeneity. These results suggest that moxibustion decreases the colonic 5-HT levels in IBS-D model rats.

#### 3.3.4 IL-1β

**FIGURE 5:**
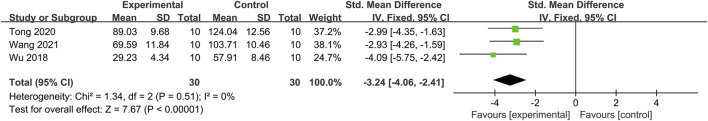
Forest plot analysis of the serum interleukin-1β concentration. CI, Confidence interval; SD, Standard deviation.

Three studies included IL-1β results (Figure 5) (Wu, 2018; Tong, 2020; Wang, 2021). The meta-analysis results indicated no heterogeneity (*I* ^*2*^ = 0%, *p* = 0.51). A fixed-effects model was used to pool the effect size, resulting in an SMD of −3.24 (95% CI: –4.06, −2.41; *p* < 0.00001), indicating a significant difference. These results demonstrate that moxibustion reduces the serum IL-1β level in IBS-D model rats.

#### 3.3.5 TNF-α

**FIGURE 6:**
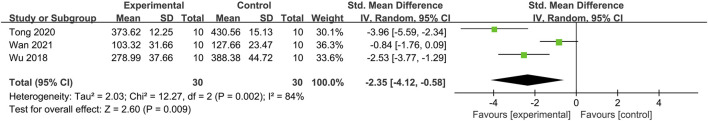
Forest plot analysis of the serum tumor necrosis factor-α concentration. CI, Confidence interval; SD, Standard deviation.

Three studies investigated the TNF-α concentration (Figure 6) (Wu, 2018; Tong, 2020; Wan, 2021). The meta-analysis showed significant heterogeneity (*I* ^*2*^ = 84%, *p* = 0.002). A random-effects model was used to pool the effect size, resulting in an SMD of −2.35 (95% CI: –4.12, −0.58; *p* < 0.00001), indicating a statistically significant difference. Using an exclusion method in the sensitivity analysis, we found that the study by Wan et al. (Wan, 2021) was the heterogeneity source, likely due to differences in the rats’ body weights. After excluding this study, the heterogeneity decreased from 84% to 47%. These results suggest that moxibustion decreases the serum TNF-α levels in IBS-D model rats.

#### 3.3.6 SP

**FIGURE 7:**
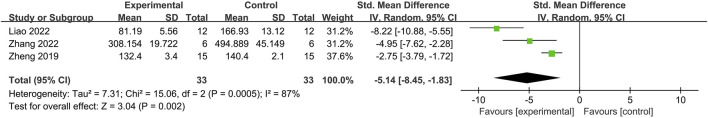
Forest plot analysis of the serum substance P concentration. CI, Confidence interval; SD, Standard deviation.

Three studies included SP results (Figure 7) (Zheng et al., 2019; Liao et al., 2022; Zhang et al., 2022). The meta-analysis showed significant heterogeneity (*I* ^*2*^ = 87%, *p* = 0.0005). A random-effects model was used to pool the effect size, resulting in an SMD of −5.14 (95% CI: –8.45, −1.83; *p* = 0.002), indicating a statistically significant difference. The sensitivity analysis did not identify the source of heterogeneity. These results indicate that moxibustion decreases the serum SP levels in IBS-D model rats.

#### 3.3.7 CGRP

**FIGURE 8:**
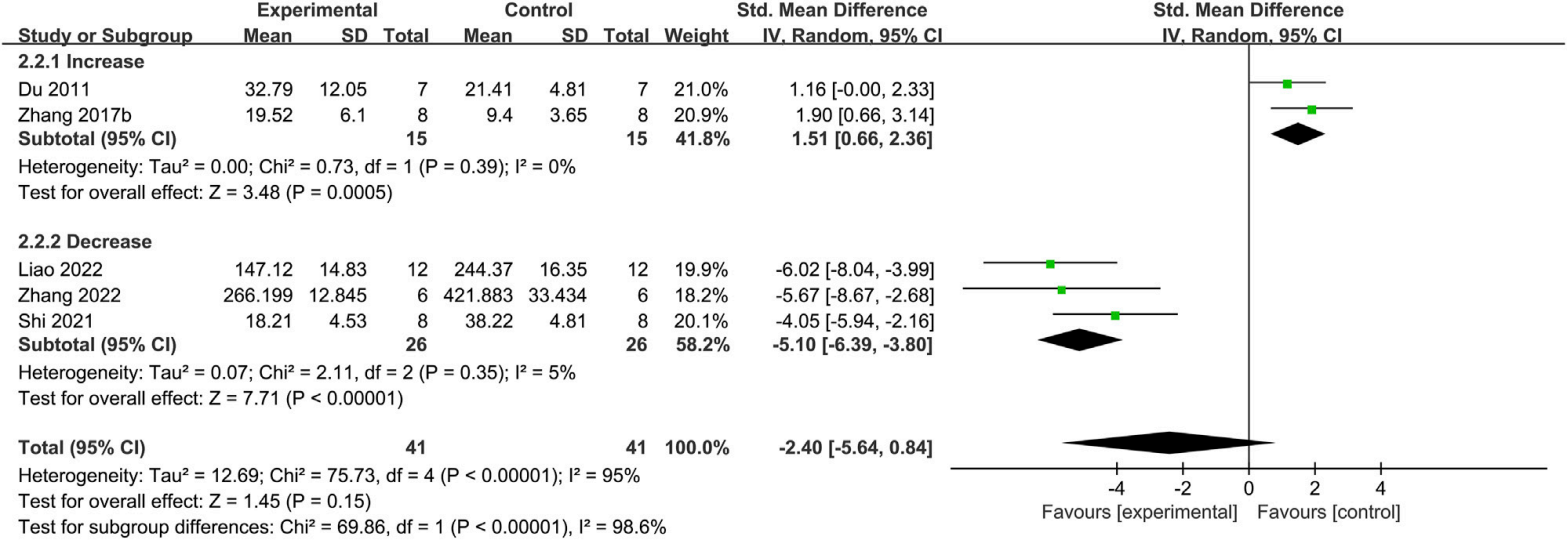
Forest plot analysis of the calcitonin gene-related peptide. CI, Confidence interval; SD, Standard deviation.

Five studies investigated the CGRP concentration (Figure 8) (Du, 2011; Zhang et al., 2017b; Shi et al., 2021; Liao et al., 2022; Zhang et al., 2022). The meta-analysis showed significant heterogeneity (*I* ^*2*^ = 95%, *p* < 0.00001). A random-effects model was used, and the results were insignificant (*p* = 0.15). Thus, moxibustion did not affect the blood CGRP levels in IBS-D model rats, and only a descriptive analysis was conducted. The effect size was not combined.

### 3.4 Publication bias

**FIGURE 9:**
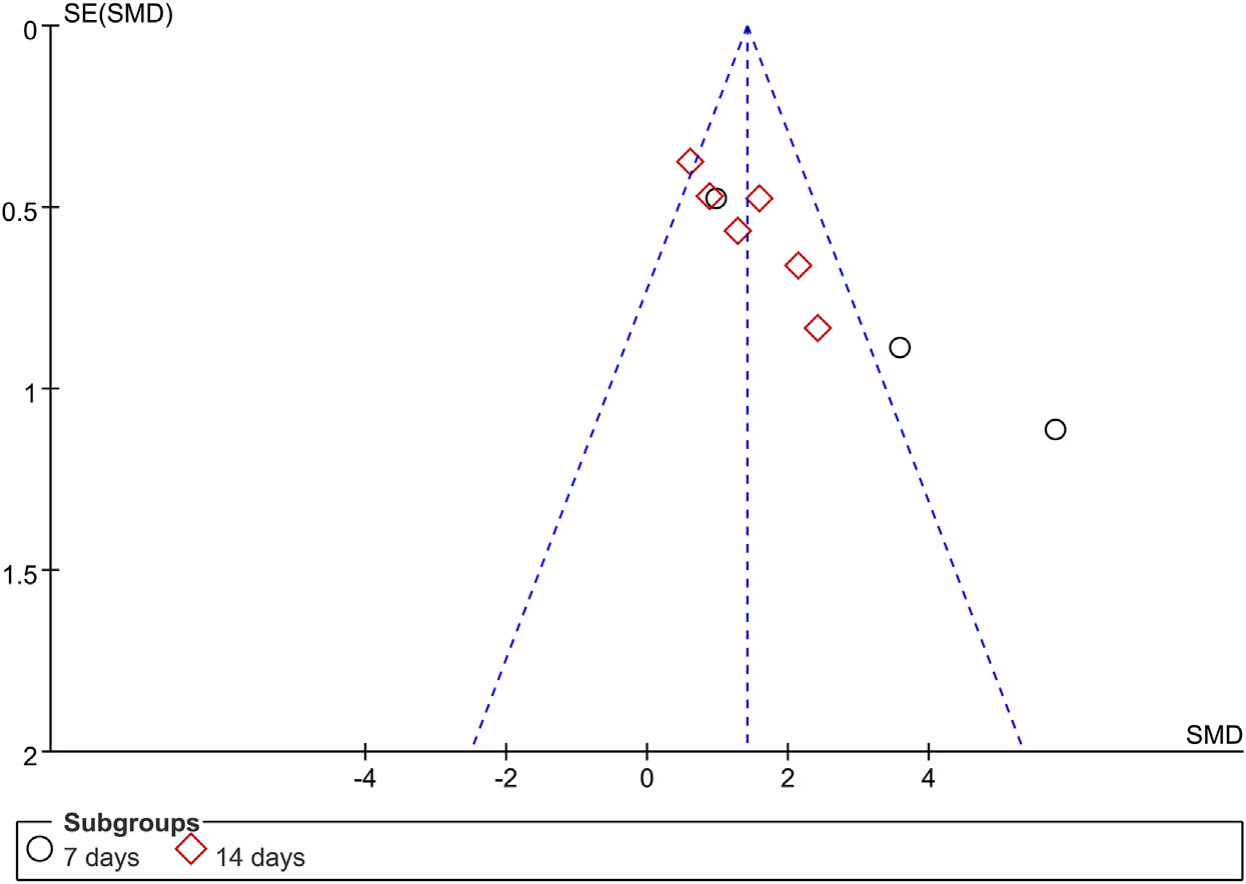
Funnel plot analysis of the threshold capacity of the abdominal withdrawal reflex. SE, Standard error; SMD, Standardized mean difference.

A funnel plot analysis was performed to assess the publication bias among the nine studies that examined the AWR threshold capacity (Figure 9). The funnel plot was asymmetric, suggesting possible publication bias among the included studies.

## 4 Discussion

Moxibustion, as a core component of traditional Chinese medicine, is one of the most widely utilized traditional medical practices globally. With its advantages of simplicity, environmental safety, and holistic regulation, moxibustion plays a crucial role in restoring the overall homeostasis of intestinal structure and function. It significantly improves the physical and mental wellbeing as well as the quality of life for patients. Particularly in the diagnosis and treatment of IBS-D, moxibustion emerges as a treatment method worthy of clinical promotion and application due to its notable benefits. Animal experiments constitute the backbone of preclinical research and are crucial for verifying the efficacy and safety of traditional Chinese medicine interventions. Employing evidence-based medicine to conduct meta-analyses of animal experiments allows for the quantitative synthesis of similar studies, significantly expanding the sample size and reducing biases and random errors among studies. This approach provides a more objective evaluation of research outcomes and enhances the reliability of the conclusions. Furthermore, systematic reviews enable the construction of a comprehensive research framework to elucidate the underlying biological mechanisms of moxibustion therapy (Zhao et al., 2018; Zhao et al., 2022). Based on these principles, our study utilized a systematic review and meta-analyses to investigate the impact of moxibustion intervention on the rate of loose stool, the AWR threshold capacity, and cytokine levels in an IBS-D rat model. We aimed to comprehensively elucidate the efficacy of moxibustion therapy in IBS-D from various perspectives and simultaneously explore its mechanisms of action, providing conclusions based on the principles of evidence-based medicine for clinical diagnosis, treatment, and fundamental research.

Visceral hypersensitivity is a primary characteristic of the IBS-D animal model. Visceral hypersensitivity refers to the heightened reactivity and sensitivity of visceral tissues to various stimuli, including physical, chemical, and psychosocial factors. It is closely associated with pain perception and involves an increased perception of physiological gastrointestinal activity and lowered thresholds for noxious stimuli, resulting in visceral hypersensitivity and reduced pain thresholds, ultimately leading to abdominal pain and triggering diarrhea. The AWR experiment, as outlined by Chen et al. (2017), involves the distension of the colon using a rectal balloon to observe the animal’s behavioral responses. This experimental approach is employed to assess the visceral sensitivity in animals and serves as an objective measure of the severity of abdominal pain. The rate of loose stool refers to the percentage of loose stools in the total fecal output of mice and can reflect the stool characteristics. Our results demonstrated that moxibustion therapy significantly improved the diarrhea rate, increased the threshold capacity for colorectal balloon distension in AWR, reduced visceral hypersensitivity, and alleviated the severity of abdominal pain compared to the IBS-D model group.

The intestinal mucosa and its secretions constitute the body’s first line of defense within the immune system, and inflammation and immune imbalance are central to the development and progression of IBS-D. IL-1β and TNF-α are crucial pro-inflammatory cytokines that contribute to the occurrence and development of IBS-D by affecting various aspects, such as disrupting the intestinal mucosal barrier, altering gastrointestinal motility, and influencing visceral sensitivity (Zhang and Fei, 2018; Sun Z. Y. et al., 2023). This study demonstrated that moxibustion therapy reduced the serum IL-1β and TNF-α levels in IBS-D model rats compared to the model group. Thus, moxibustion therapy may suppress intestinal inflammation by regulating the release of inflammatory factors IL-1β and TNF-α in the serum. These events, in turn, protect the intestinal mucosal barrier by maintaining its permeability and alleviating inflammation, ultimately ameliorating stool-related symptoms in IBS-D model rats.

The brain-gut axis represents a bidirectional regulatory neuro-endocrine-immune network within the brain-gut interaction. Neuropeptides, known as brain-gut peptides, are distributed in the gastrointestinal tract and the central nervous system and are essential mediators in facilitating this brain-gut interaction (Sun et al., 2022). Presently, over 60 brain-gut peptides have been discovered, and 5-HT, SP, and CGRP have been closely associated with IBS-D. 5-HT, also known as serotonin, is a critical neurotransmitter in the brain-gut axis linked to the development of neuropsychiatric disorders, such as anxiety and depression. Approximately 95% of the body’s 5-HT is synthesized in enterochromaffin cells within the intestinal tract, influencing brain activity, pain perception, gastrointestinal motility, mucosal inflammation, and immune responses via the gut-brain axis. Elevated 5-HT levels affect the regulation of the vagus nerve, leading to heightened visceral sensitivity (Chen B. W. et al., 2021). SP is an 11-amino acid brain-gut peptide in the nervous system and gastrointestinal tract that helps transmit and modulate pain by transmitting sensory nerve impulses to the central spinal cord; it also promotes smooth muscle contractions, causing abnormal gastrointestinal hyperactivity and increased frequency of diarrhea (Lu et al., 2015). CGRP is an important neuropeptide primarily found in visceral nerves, vagal afferent fibers, and intramural nerve plexuses in the gastrointestinal tract and is involved in the formation of visceral sensitivity (Fang et al., 2017; Ni et al., 2022). This study demonstrated that moxibustion therapy significantly reduced the activity of colon 5-HT in IBS-D model rats compared to the model group. In addition, it inhibited the harmful effects of SP, a pain-inducing substance, on the gastrointestinal tract, suppressing pain signal transmission. This intervention also alleviated visceral hypersensitivity, ultimately modulating the mental and activity states of the IBS-D model rats. CGRP is widespread throughout the body, and its diverse biological effects are contingent upon its binding to various receptors. Notably, this study indicated that the effects of moxibustion therapy on CGRP levels in IBS-D remain unclear and necessitate further investigation.

We used the SYRCLE tool to assess the bias risk in the included studies, finding that all included studies mentioned randomization methods, but specific details regarding the randomization process were not provided. We recommend explicitly describing the methods of sequence generation, such as computer-generated random numbers or the use of random number tables. Regarding baseline characteristics, IBS-D models were induced in six studies before random allocation, which helped achieve balanced baseline characteristics among groups. However, for the remaining studies where disease induction occurred after randomization, there was insufficient information regarding the blinding of personnel involved in implementing the intervention measures, leading to uncertainty regarding their bias risk. Another potential source of bias was allocation concealment, where the included literature had inadequate reporting, making it impossible to determine whether concealed grouping was implemented. Additionally, attention should be paid to the unit of analysis. Although most studies did not describe whether outcome assessors were blinded, it did not affect our study’s measurement of objective outcome indicators. Through this systematic review, we observed that some of the items evaluated by the SYRCLE tool lacked detailed information in certain publications, indicating room for improvement in reporting quality. Therefore, it is essential for future research to conduct scientifically rigorous experiments and adhere to reporting standards for high-quality animal experimental studies.

This study identified significant heterogeneity in the AWR threshold values. A subsequent heterogeneity analysis revealed that the duration of moxibustion treatment affected the AWR threshold values. However, the specific extent of this impact requires further investigation. Additionally, body weight disparities among the rats contributed to the heterogeneity of the TNF-α results. Thus, we recommend utilizing male Sprague-Dawley rats with an approximate body weight of 200 g for IBS-D research. In terms of moxibustion intervention parameters, we suggest a suspension height of 2–3 cm, an intervention duration of 30–40 min, and a recommended treatment course of 14 days.

A systematic safety assessment revealed that three rats died during the model replication process in the study by Liao et al. (2022), and two rats accidentally died from pneumothorax during the modeling period in the study by Li (2022). However, during the moxibustion intervention, fatalities did not occur. Furthermore, compared to the model group, rats subjected to moxibustion generally exhibited favorable conditions, including stool consistency, fur appearance, and mental and activity status, suggesting that moxibustion safely and effectively alleviates symptoms, such as abdominal pain and diarrhea in the IBS-D model rats.

Limitations of this study include the restriction to studies published in Chinese and English, which may introduce language and publication bias. Some studies presented data in image format, and despite our attempts to contact the corresponding authors, only one study provided the necessary data; data from the remaining studies could not be obtained and were consequently excluded from this analysis. Additionally, the heterogeneity observed in certain outcome measures can be attributed to variations in factors, such as the choice of animal species, the standards and methods of IBS-D induction, experimental conditions and standards, types of moxibustion used, intervention duration, and the selection of moxibustion points. These discrepancies can potentially diminish the overall relevance of the animal models employed in this study.

In conclusion, the current evidence suggests that moxibustion therapy modulates the gut-brain interaction through multiple pathways and targets. It also reduces visceral hypersensitivity, suppresses intestinal inflammation, and regulates immune balance, improving the rate of loose stool and enhancing the AWR threshold capacity, resulting in effective pain relief and antidiarrheal effects. However, due to limitations in the quantity and quality of the included studies, these conclusions require further validation through additional high-quality research.

## Data Availability

The original contributions presented in the study are included in the article/Supplementary Material, further inquiries can be directed to the corresponding authors.
