# Supplementary material for: Efficacy of moxibustion in diarrhea-predominant irritable bowel syndrome model rats: a systematic review and meta-analysis
Source: Front Bioeng Biotechnol. 2023 Dec 15;11:1309661. doi: 10.3389/fbioe.2023.1309661 (PMC10757337; doi:10.3389/fbioe.2023.1309661)
Supplement: Supplementary file 1 [file DataSheet1.PDF]

Supplementary material: search strategy

1.PubMed

("moxibustion"[MeSH Terms] OR "moxibustion"[Title/Abstract] OR "separated moxibustion"[Title/Abstract] OR "heat-sensitive point moxibustion"[Title/Abstract]) AND ("irritable bowel syndrome"[MeSH Terms] OR "irritable bowel syndrome"[Title/Abstract] OR "IBS"[Title/Abstract] OR "spastic colon"[Title/Abstract] OR "irritable colon"[Title/Abstract] OR "irritable bowel"[Title/Abstract] OR "functional bowel"[Title/Abstract] OR "colonic disease"[Title/Abstract] OR "gastrointestinal syndrome"[Title/Abstract] OR "gastrointestinal syndromes"[Title/Abstract]) AND ("rats"[MeSH Terms] OR "rats"[Title/Abstract])

2.EMBASE

('irritable colon'/exp OR ('spastic colon':ti,ab,kw OR 'irritable colon':ti,ab,kw OR 'irritable bowel':ti,ab,kw OR 'functional bowel':ti,ab,kw OR 'colonic disease':ti,ab,kw OR 'gastrointestinal syndrome':ti,ab,kw OR 'gastrointestinal syndromes':ti,ab,kw OR ibs:ti,ab,kw OR 'irritable bowel syndrome':ti,ab,kw)) AND ('moxibustion'/exp OR (moxibustion:ti,ab,kw OR 'separated moxibustion':ti,ab,kw OR 'heat sensitive point moxibustion':ti,ab,kw)) AND ('rat'/exp OR rat:ti,ab,kw)

3.Web of Science

TS=Irritable Bowel Syndrome AND TS=Rats AND TS=Moxibustion

4.China National Knowledge Infrastructure

(SU=’肠易激’ OR SU=’IBS’) AND SU=’大鼠’ AND (SU=’灸’ OR SU=’隔物灸’ OR SU=’热敏灸’ OR SU=’雷火神针’ OR SU=’太乙神针’)

5. Wanfang Database

(主题:(IBS) or 主题:(肠易激综合征)) and 主题:(大鼠) and (主题:(灸) or 主题:(雷火神针) or 主题:(太乙神针))

6. VIP Database

(U=(IBS) OR U=(肠易激)) AND (U=(大鼠)) AND (U=(灸) OR U=(雷火神针) OR U=(太乙神针))
